# Intensity-modulated radiotherapy for hepatocellular carcinoma: dosimetric and clinical results

**DOI:** 10.18632/oncotarget.19219

**Published:** 2017-07-13

**Authors:** Sun Hyun Bae, Won Il Jang, Hee Chul Park

**Affiliations:** ^1^ Department of Radiation Oncology, Soonchunhyang University College of Medicine, Bucheon, Korea; ^2^ Department of Radiation Oncology, Korea Institute of Radiological and Medical Sciences, Seoul, Korea; ^3^ Department of Radiation Oncology, Samsung Medical Center, Sungkyunkwan University School of Medicine, Seoul, Korea; ^4^ Department of Medical Device Management and Research, SAIHST, Sungkyunkwan University, Seoul, Korea

**Keywords:** conformal radiotherapy, hepatocellular carcinoma, intensity-modulated radiotherapy, radiotherapy

## Abstract

Since the introduction of 3-dimensional conformal radiotherapy (3DCRT), new radiotherapy techniques have expanded the indication of radiotherapy for the treatment of hepatocellular carcinoma (HCC), from the hitherto palliative to a now curative-intent purpose. Intensity-modulated radiotherapy (IMRT), currently the most advanced radiotherapy technique, is considered an attractive option for the treatment of HCC, and is more widely applied because it can deliver a higher dose to the tumor than 3DCRT while sparing surrounding normal organs. However, the advantages and potential disadvantages of IMRT for treating HCC have not been fully established. This article deals with three different IMRT techniques, including static IMRT and volumetric modulated arc therapy using conventional multileaf collimator (MLC) mounted linear accelerators, and helical IMRT using binary MLC mounted helical tomotherapy machine. We review dosimetric and clinical studies for these IMRT techniques for the treatment of HCC.

## INTRODUCTION

Historically, the role of radiotherapy (RT) for the treatment of hepatocellular carcinoma (HCC) has been limited because of the low tolerance of the whole liver to RT and the risk of radiation-induced liver disease (RILD) [1–3]. With the introduction of 3-dimensional conformal radiotherapy (3DCRT), however, the delivery of conformal partial liver RT allows for safe dose escalation as the liver parenchyma is arranged with a functionally parallel architecture [4, 5]. In addition, further development of radiotherapy techniques, including intensity-modulated radiotherapy (IMRT) and stereotactic body radiotherapy (SBRT), has expanded the indication of RT for the treatment of HCC from a palliative to a curative-intent aim [6–9]. The current practical guidelines for the treatment of HCC are summarized in Table 1 [10–17]. Although the use of RT has been limited in several guidelines, the recommendation of RT as a local treatment modality from recent versions of the National Comprehensive Cancer Network (NCCN) guidelines [17] and the practice guidelines from the Korean Liver Cancer Study Group and National Cancer Center Korea [11], is noteworthy changes. Especially, the NCCN guidelines suggest IMRT is helpful in selected HCC patients. However, there is insufficient evidence to support the routine use of RT as shown in Table 1. The level of evidence regarding RT use is only B-C. Therefore, further efforts are needed to maximize the efficacy and minimize the toxicity of RT. The solution might be provided by the application of IMRT, which is the most advanced RT technique currently available.

**Table 1:** Current practical guidelines for the management of hepatocellular carcinoma (HCC) around the world

Region	Group	Abbreviations	Publishing year	Comments about RT
Asia	Korean Liver Cancer Study Group and the National Cancer Center [11]	KLCSG-NCC	2014	· EBRT can be performed in HCC patients if liver functions are CP class A or superb B and the irradiated total liver volume receiving ≥ 30 Gy is ≤ 60% (B1^a)^). · EBRT can be considered for HCC patients ineligible for surgical resection, LT, RFA, PEIT, or TACE (C1). · EBRT can be considered for HCC patients who show incomplete response to TACE when the dose-volume criteria in Recommendation 1 are met (B2). · EBRT can be considered for HCC patients with PVTT when the dose-volume criteria in Recommendation 1 are met (C1). · EBRT is performed to alleviate symptoms caused by primary HCC or its metastases (B1).
	Japan Society of Hepatology [12]	JSH	2013	· 3DCRT can be considered for patients with PVTT or unresectable tumors who are contraindicated for other standard treatment methods because of complications or other reasons (C1). · There is insufficient scientific evidence to support the extension of survival duration by RT alone. However, survival duration is expected to increase in unresectable cases if RT is combined with TACE (C1).
	Asian Pacific Association for the Study of the Liver [13]	APASL	2010	None
Europe	European Association for the Study of the Liver and the European Organization for Research and Treatment of Cancer [14]	EASL–EORTC	2012	· 3DCRT is under investigation, and there is no evidence to support this therapeutic approach in the management of HCC (2C)
	European Society for Medical Oncology - European Society of Digestive Oncology [15]	ESMO-ESDO	2012	· EBRT can be used to control pain in patients with bone metastases [II, B].
USA	American Association for the Study of Liver Disease [16]	AASLD	2010	· There are multiple other treatment modalities such as octreotide, interferon, EBRT, tamoxifen, or anti-androgenic therapy, but none have been shown to improve survival
	National Comprehensive Cancer Network (NCCN) Clinical Practice Guidelines in Oncology (NCCN Guidelines^®^) Hepatobiliary Cancers (Version 1.2016)] [17]	NCCN	2016	· All tumors irrespective the location may be amenable EBRT (SBRT, IMRT, or 3DCRT). · There is growing evidence for the usefulness of SBRT in the management of patients with HCC. SBRT can be considered as an alternative to the ablation/embolization techniques mentioned above or when there therapies have failed or are contraindicated. · Proton beam therapy may be appropriate in specific situations. · Palliative EBRT is appropriate for symptom control and/or prevention of complications from metastatic HCC lesions, such as bone or brain.

RT: radiotherapy, EBRT: external beam radiotherapy, CP: Child Pugh, LT: liver transplantation, RFA: radiofrequency ablation, PEIT: percutaneous ethanol injection therapy, TACE: transarterial chemoembolization, PVTT: portal vein tumor thrombosis, 3DCRT: 3-dimensional conformal radiotherapy, SBRT; stereotactic body radiotherapy, IMRT: intensity-modulated radiotherapy.

^a)^ the alphabet means the level of evidence and the number means the grade of recommendation. The definition of grading system in detail should be checked at each guideline.

Compared with previous treatment techniques such as 3DCRT, the features of IMRT are the inverse treatment planning process and the use of large number of treatment fields or subfields that fall within the conformal beam’s-eye view of the target [18]. Therefore, IMRT provides a high precision and an exquisitely conformal dose distribution using multiple beams, each with a non-uniform intensity profile [19, 20]. Conventional multileaf collimator (MLC)-mounted linear accelerators are equipped with the following three types of IMRT delivery systems that enable the creation of non-uniform intensity profiles: (1) step-and-shoot IMRT, in which small MLC-generated segments are used, and radiation is not delivered while the leaves move to create the next segment, (2) sliding window IMRT, in which modulated MLC velocity in multiple static radiation fields is used, and the radiation is delivered as the leaves are moving, and (3) volumetric modulated arc therapy (VMAT), which is a rotational form of IMRT whereby moving MLC and changing dose delivery rates occur during rotation [21]. On the other hand, helical tomotherapy is an independent rotational IMRT machine that has a binary MLC to quickly open and close apertures in front of the different beam elements in the fan beam. It uses a slow, continuous movement of the patient support system with a rapidly rotating X-ray source to make many rotations possible in a relatively short period of time using a helical dose delivery technique similar to the modern spiral CT scanner [18].

Currently, IMRT is postulated to be an attractive technique for the treatment of HCC, and is becoming more widely applied [22]. Although intrafractional and interfractional setup error from the effects of respiratory motions may introduce dosimetric errors, the application of 4-D CT, advanced immobilization system, use of gating and tracking techniques, and fractionation schedule could minimize physically treatment inaccuracy [23–27]. On the other hand, the clinical advantages and potential disadvantages of IMRT for treating HCC have not been fully established. This article reviews dosimetric and clinical studies using IMRT for the treatment of HCC. To compare the characteristics of IMRT techniques, we define the step-and-shoot and sliding window techniques as static IMRT (s-IMRT), the rotational IMRT using conventional MLCs as VMAT, and the rotational IMRT using helical tomotherapy as helical IMRT (h-IMRT).

## DOSIMETRIC STUDIES

### Target volume coverage

When the conformity index (CI) and homogeneity index (HI) were used to assess target volume coverage, Lee et al. [28] reported significantly improvement in both parameters with h-IMRT compared with s-IMRT and 3DCRT for 12 patients with advanced HCC. Chen et al. [29] showed that VMAT and s-IMRT achieved significantly better CI than 3DCRT. Additionally, VMAT showed significant improvement compared with s-IMRT and 3DCRT with regard to HI. To compare among IMRT techniques, Hsieh et al. [30] made h-IMRT and s-IMRT plans in nine HCC patients with portal vein tumor thrombosis (PVTT). CI values showed no significant difference between h-IMRT and s-IMRT, but HI values significantly improved with h-IMRT compared with s-IMRT. Park et al. [31] reported that the CI of VMAT was significantly better than that of s-IMRT. Another study showed no significant differences in CI values between h-IMRT and s-IMRT, however h-IMRT achieved significantly better HI values than s-IMRT [32].

### Sparing of the liver

The liver sparing effect between 3DCRT and IMRT techniques is affected by tumor location and size. Lee et al. [28] analyzed the dosimetric parameters of normal liver based on the location of HCC lesion (right lobe vs. left lobe vs. both lobes). The high dose region, which included the volume of the normal liver receiving more than 40 Gy (V40), V50, and V60, was significantly smaller in h-IMRT than s-IMRT and 3DCRT. On the other hand, the low dose region (V20) and mean dose were significantly smaller in 3DCRT than h-IMRT and s-IMRT. These differences were meaningful when the tumor was present in a single lobe, either right or left, but disappeared when the tumor was located in both lobes. Cheng et al. [29] compared liver protection, based on tumor size in 20 patients with centrally located HCC on the right lobe. The mean dose of the normal liver was significantly higher using s-IMRT than 3DCRT and VMAT. The low dose region, including V5 and V10, was significantly smaller in 3DCRT, and the high dose region, including V20 and V30, was significantly smaller in VMAT. In larger HCC (> 8 cm), the mean dose was lower in 3DCRT than s-IMRT and VMAT, leading the authors to suggest that 3DCRT might be a more suitable technique for larger tumors (> 8 cm) located in the right lobe, in terms of minimizing the risk of RILD. This size limitation of IMRT to achieve a maximal liver sparing effect was supported by another study that compared s-IMRT and proton beam therapy in 10 HCC patients [33]. Using the Lyman-normal-tissue complication probability model to estimate the risk of RILD, the risk of RILD for s-IMRT dramatically increased between nominal diameters of 6.3–7.8 cm gross tumor volume.

Among the IMRT techniques, the mean and low dose region of the normal liver in h-IMRT and VMAT were higher than s-IMRT [28, 30, 31, 34]. To minimize this difference, one study performed h-IMRT re-planning [32]. Specifically to reduce V15 of the normal liver as much as possible, which was identified as the best dosimetric parameter for predicting RILD in their previous study, the mean dose of the total liver and V15 of the normal liver were still significantly higher in h-IMRT than s-IMRT [35]. Another method to improve liver sparing in h-IMRT is to apply a directional beam blocking technique, especially for left lobe lesions [28]. This technique reduces the normal liver dose similar to s-IMRT, although homogenous dose distribution within the tumor slightly deteriorates. In case of VMAT, the use of non-coplanar arcs provides superior liver protection than s-IMRT for HCC located in the left lobe [36]. Because of the asymmetric shape and eccentric location of the liver, non-coplanar arcs from the right cranial direction may focus more radiation on the left lobe, and threfore reduce the radiation dose to the normal liver.

Despite many dosimetric studies, it is unclear whether the increase of the low dose region in the normal liver using IMRT techniques, compared to 3DCRT, could increase the risk of RILD. From a radiobiology perspective, the liver is the classic example of a parallel architecture model [37]. However, although RILD mainly occurs if the partial volume damaged exceeds a threshold, the risk of RILD is also added by dose distribution of the functional reserve and subunit radiosensitivity [38, 39]. As a result, the increased risk of RILD associated with IMRT may be related to dose distribution throughout the liver and the patients’ functional reserve, rather than the maximum dose to a limited area [40]. This is supported by evidence from the lung, another typical example of a parallel architecture model, where an increase in radiation pneumonitis is seen when there is an increase to the low dose region during IMRT. An extra dose constraint for the low dose region (V5), in addition to V20 and the mean dose significantly reduced radiation pneumonitis after IMRT in locally advanced non-small cell lung cancer [41, 42]. Therefore, further studies are needed to evaluate the potential risk of RILD in the low dose region associated with IMRT in HCC patients.

### Sparing of other organs at risk (OARs) except the liver

Kuo et al. [34] reported that mean doses of the stomach and left kidney were significantly lower using s-IMRT than 3DCRT in nine patients with unresectable HCC. However, there was no difference between 3DCRT and VMAT. Lee et al. [28] found that the best sparing of high dose regions in the stomach and small bowel (V40, V50, and V60) were achieved with h-IMRT, followed by s-IMRT, and 3DCRT without statistical significance. In HCCs located in both lobes, however, s-IMRT showed better sparing of the stomach than h-IMRT. For right-lobe HCC, irradiation dose to the left kidney was higher with h-IMRT because of the helical delivery nature. Park et al. [31] also suggested that VMAT tended to be more effective in sparing non-liver OARs than s-IMRT. For patients with primary HCC and PVTT, V35 of the duodenum in VMAT was significantly smaller than that in s-IMRT. For patients with primary HCC alone, V20 of the kidney was significantly smaller in VMAT than s-IMRT. The maximal point dose of the spinal cord was significantly lower in VMAT than s-IMRT regardless of tumor location.

The prevalence of gastric ulcer in cirrhotic patients was as high as 20%, compared to 2–4% in the general population because of underlying cirrhosis and portal hypertensive gastropathy (PHG) [43, 44]. The prevalence of gastroduodenal toxicity in HCC patients who received 3DCRT and underwent endoscopy was 30–50% [45–47]. Until now, we cannot determine whether the gastroduodenal toxicities developed as a consequence of RT or occurred spontaneously due to cirrhosis and PHG. Severe hematemesis by aggravation of pre-existing PHG was reported in HCC patients receiving 3DCRT regardless of radiation dose [48]. Considering that V25 or V35 of gastroduodenum were significant dosimetric parameters affecting severe gastroduodenal toxicity in two studies, the application of IMRT would reduce gastroduodenal toxicity in HCC patients by sparing the high dose regions of the gastroduodenum as mentioned above [45, 47].

The strengths and weakness of IMRT techniques are summarized in Table 2.

**Table 2:** The strengths and weakness of intensity-modulated radiotherapy (IMRT) techniques for hepatocelluar carcinoma

	s-IMRT	h-IMRT	VMAT
Strength	*Compared with 3DCRT* - Improving target coverage - Sparing OARs	-	-
	*Compared with h*-*IMRT and VMAT* - Sparing the normal liver	*Compared with s*-*IMRT* - Same or better homogeneous dose distribution within target - Sparing non-liver OARs	*Compared with s*-*IMRT* - Same or better homogeneous dose distribution within target - Sparing non-liver OARs - Lower MUs - Shorter treatment time: reduction of intra-fractional movement; improvement of patient's comfort; higher patient throughput
Weakness	*Compared with 3DCRT* - Higher MUs - Longer treatment time - Larger low dose region of OARs - Less sparing the normal liver in case of large tumor > 6–8 cm	-	-
	*Compared with h*-*IMRT or VMAT* - More dependent on the beam angle and the experience of the physicist	*Compared with s*-*IMRT* - Larger low dose region of the normal liver (consider a directional block)	*Compared with s*-*IMRT* - Larger low dose region of the normal liver (consider use of non-coplanar arc) - Limitation of non-coplanar arc: availability of only asymmetric partial arc; Decrease of advantage duo to increased treatment time by couch rotation and increased MUs

s-IMRT: static IMRT using step-and-shoot technique and sliding window technique delivered by conventional multileaf collimator (MLC)-mounted linear accelerators, h-IMRT: helical IMRT using rotational dose delivery by binary MLC mounted helical tomotherapy, VMAT: volumetric modulated arc therapy using rotational dose delivery by conventional MLC mounted linear accelerators, 3DCRT: 3-dimensional conformal radiotherapy, OARs: organs at risk, MUs: monitor units.

## CLINICAL STUDIES

### 3DCRT vs. IMRT

There were two clinical studies that compared 3DCRT to IMRT for the treatment of HCC, and both used h-IMRT technique (Table 3). Yoon et al. [49] retrospectively reviewed 187 patients with locally advanced HCC treated with RT. Sixty-five patients received h-IMRT (median fractional dose and total dose; 2.5 Gy and 50 Gy) and 122 patients received 3DCRT (median fractional dose and total dose; 1.8 Gy and 45 Gy). Baseline characteristics were not significantly different between the two groups. With median follow-up of 21 months, the local control rate (LCR) and overall survival (OS) rate at 3 years were significantly different according to RT techniques; the LCRs were 28% and 47% (*P* = 0.007) for 3DCRT and h-IMRT, respectively, and the OS rates were 14% and 33% (*P* < 0.001) for 3DCRT and h-IMRT, respectively. The use of h-IMRT was the significant parameter for OS in both univariate and multivariate analyses. On the other hand, RILD or severe toxicity ≥ grade 3 according to the National Cancer Institute Common Terminology Criteria for Adverse Events (CTCAE) was not different. The authors suggested that delivery of high doses without increased toxicity, as achieved with the use of h-IMRT led to improve LCR, leading to a longer OS. A recent study also reported the efficacy of h-IMRT [50]. They compared 3DCRT to h-IMRT in 118 advanced HCC patients with PVTT and/or inferior vena cava tumor thrombosis (IVCTT). Fifty-four patients received h-IMRT (median total dose; 60 Gy) and 64 patients received 3DCRT (median total dose; 54 Gy). The overall objective response rate (RR), including complete response and partial response, was 43% in the 3DCRT group and 70% in the h-IMRT group (*P* = 0.056). When the tumor thrombi were assessed, the objective RR was significantly different, being 47% after 3DCRT and 67% after h-IMRT (*P* = 0.031). With median follow-up of 11.8 months, the OS rate at 1 year was 36% in patients who received 3DCRT and 59% in patients who received h-IMRT (*P* = 0.005). Severe toxicity ≥ grade 3 as defined by the Radiation Therapy Oncology Group (RTOG) toxicity criteria was not different. The authors suggested that the better RR after h-IMRT, especially for the tumor thrombi, allowed more patients to receive further trans-arterial chemo-embolization (TACE) after RT, and both increased RR and additional treatments improved survival.

**Table 3:** Clinical studies compared 3-dimensional conformal radiotherapy (3DCRT) with helical intensity-modulated radiotherapy (h-IMRT) by helical tomotherapy for hepatocellular carcinoma

Studies	RT technique	No. of patients	CP class	Tumor size	VI	RT dose	Combined Treatment	Median f/u (mo)	LCR	OS	Toxicity
Yoon 2014 [49]	3DCRT	122	A	Median 10 cm (1–18.6)	PVTT in 79%	1.8–5 Gy/fx, 36–60 Gy	CCRT with HAIC in 95.2%	21	28% at 3 yrs	14% at 3 yrs	RILD in 5%
	h-IMRT	65		Median 9 cm (2.2–18.8)	PVTT in 82%	2.5–3.5 Gy/fx, 47.5–60 Gy			47% at 3 yrs (*P =* 0.007)	33% at 3 yrs (*P <* 0.001)	RILD in 3% (NS)
Hou 2016 [50]	3DCRT	64	A: 50 B: 14	Mean 8.6 cm	PVTT in 88%; IVCTT in 12%	1.8–2 Gy/fx, 40–60 Gy	None	11.8	43%^a)^	36% at 1 yr	Gr 3 toxicity^b)^ in 5%
	h-IMRT	54	A: 46 B: 8	Mean 7.5 cm	PVTT in 89%; IVCTT in 11%	2.5–4 Gy/fx, 40–66 Gy			70%^a)^	59% at 1 yr (*P =* 0.005)	Gr 3 toxicity^b)^ in 2% (NS)

CP class: Child-Pugh class, VI: vascular invasion, LCR: local control rate, OS: overall survival rate, PVTT: portal vein tumor thrombosis, CCRT: concurrent chemo-radiotherapy, HAIC: hepatic arterial infusion chemotherapy, RILD: radiation-induced liver disease, IVCTT: inferior vena cava tumor thrombosis, NS: no significant.

^a)^means overall response rate.

^b)^is classified by the grading system of the Radiation Therapy Oncology Group.

### IMRT studies

Several studies have reported treatment outcomes after IMRT for HCC, as shown in Table 4 [51–59]. The objective RR ranged from 43% to 74%, and the OS rate at 1 year ranged from 45% to 85%. The diversity of treatment outcomes among the IMRT studies may be mainly due to differences in baseline characteristics of the patients. Although all studies included patients with Child-Pugh (CP) class B and patients with PVTT, the proportion of these patients were different among the individual studies, and it has been well documented that they are important prognostic factors for survival [60–62]. Kang et al. [51] found that CP class and PVTT were significant parameters affecting RR after s-IMRT. And objective response was the only significant parameter affecting OS in a multivariate analysis. Kong et al. [55] found that CP class and PVTT were significant parameters for OS on multivariate analysis. Kim et al. [56] reported that CP class was a significant parameter that predicted the response of PVTT, and involvement of the main portal trunk was a significant parameter for an unfavorable OS. In terms of dose-response relationship, only one study showed that a higher biologically effective dose > 65.5 Gy10 contributed significantly to superior local control, both in univariate analysis and multivariate analyses [57]. On the other hand, other studies have not verified the association between RT dose and RR or survival [54–56].

**Table 4:** Clinical studies treated with intensity-modulated radiotherapy (IMRT) for hepatocellular carcinoma

Studies	IMRT technique	No. of pts	CP class	Tumor size	VI	RT dose	Combined Treatment	Median f/u (mo)	RR	OS	Toxicity
Kang 2011 [51]	s-IMRT	27	A: 19 B: 8	Mean 11.4 cm (8.1–18.2)	PVTT in 63%, IVCTT in 4%	1.8 Gy/fx, 45–64.8 Gy	CCRT with HAIC in 11%; TACE during RT in 11%	5	44%	Median survival = 5 mo	Hepatic toxicity of Gr 3 in 7% and Gr 5 in 7%a) (CTCAE)
Zhang 2016 [52]	s-IMRT	54	N/A	Median 6.3 cm (1.2–14.0)	PVTT in 48%	1.8–2 Gy/fx 44–70 Gy	TACE	29	65%	85% at 1 yr and 50% at 2 yrs	No RILD; Hepatic toxicity of Gr 3 in 6%
McIntoch 2009 [53]	h-IMRT	20	A: 11 B: 9	Mean 9.3 cm (1.3–17.4)	PVTT in 40%	2.5 Gy/fx, median 50 Gy	CCRT with capecitabine	N/A	66%	75% at 1 yr and 50% at 2 yrsb)	No increase more than Gr2 from baseline (CTCAE)
Chi 2010 [54]	h-IMRT	23	A: 15 B: 8	N/A	PVTT in 22%	2.5–4.5 Gy/fx, median 52.5 Gy	CCRT with sunitinib	16	74%	70% at 1 yr	Hepatitis of Gr3 in 4%; GI bleeding of Gr3 in 9% (RTOG)
Kong 2013 [55]	h-IMRT	22	A: 15 B: 7	Median 4.4 cm (0.9–16.4)	PVTT in 36%	1.8–4 Gy/fx, 30–60 Gy	None	14.4	73%	86% at 1 yr and 69% at 2 yrs	RILD in 5%; No severe toxicity ≥ Gr 3 (CTCAE)
Kim 2013 [56]	h-IMRT	35	A: 28 B: 7	N/A	PVTT in 100%	4.5–6 Gy/fx, 45–60 Gy	CCRT with capecitabine	12.9	43% for PVTT; 52% for primary HCC	51% at 1yr and 22% at 2 yrs	No RILD; CP class deterioration in 34%c); Late GI toxicity of Gr 3 in 6% (RTOG)
Huang 2015 [57]	h-IMRT	38	A: 27 B: 11	Median 4.6 cm (2.5–16.7)	PVTT in 63%	1.8–2.4 Gy/fx, 46–71.8 Gy	None	17.2	53%	56% at 1 yr and 32% at 2 yrs	RILD in 3%; Hepatic toxicity ≥Gr 3 in 13%; Late GI toxicity of Gr 3 in 3% (RTOG)
Son 2017 [58]	h_IMRT	56	A: 47 B: 9	329.5 ± 271.5 mLd)	PVTT in 55%	4–5 Gy/fx, 40–50 Gy	None	13	N/A	52% at 1 yr and 23% at 2 yrs	N/A
Wang 2013 [59]	VMAT	138	A: 96 B: 42	Mean 583 mLd) (22.9–3262.7)	PVTT in 54%	1.8–2 Gy/fx, 45–66 Gy	None	9	64%e)	45% at 1 yr	Nonclassic RILD in 13%

CP class: Child-Pugh class, VI: vascular invasion, RR; response rate including complete response and partial response, OS: overall survival rate, s-IMRT: static IMRT using step-and-shoot technique and sliding window technique delivered by conventional multileaf collimator (MLC)-mounted linear accelerators, h-IMRT: helical IMRT using rotational dose delivery by binary MLC mounted helical tomotherapy, VMAT: volumetric modulated arc therapy using rotational dose delivery by conventional MLC mounted linear accelerators, PVTT: portal vein tumor thrombosis, IVCTT: inferior vena cava tumor thrombosis, CCRT: concurrent chemo-radiotherapy, HAIC: hepatic arterial infusion chemotherapy, TACE: transarterial chemo-embolization, CTCAE: the National Cancer Institute Common Terminology Criteria for Adverse Events, N/A: not available, RTOG; the Radiation Therapy Oncology Group toxicity criteria, RILD: radiation-induced liver disease.

^a)^Fatal hepatic toxicity occurred only in patients who received combine treatment: 1 patient received TACE and 1 patient received HAIC.

^b)^was estimated in patients with CP A class.

^c)^12 patients (34%) experienced CP class deterioration: 2 patients experienced local tumor progression and 2 patients had the progression of distant metastases.

^d)^means planning target volume.

^e)^Percentage are relative to 109 patients with available follow-up image.

Table 4 also shows IMRT-related toxicity. Combined treatment tends to increase severe toxicity. Kang et al. [51] reported two fatal hepatic toxicities (grade 5 according to CTCAE). Both patients received combined treatments; one patient started s-IMRT 10 days after TACE, and the other patient received concurrent chemoradiotherapy (CCRT) with hepatic arterial infusion chemotherapy (HAIC). They recommended that special attention should be given to any other treatment given during or immediately after IMRT. One study concurrently used sunitinib during IMRT and reported the highest rate of gastrointestinal (GI) bleeding (9%) [54]. Sunitinib is a multityrosine kinase inhibitor, and one of the vascular endothelial growth factor inhibitors (VEGFIs). It is considered experimentally as a radiosensitizer under clinical setting [63]. Although the authors did not suggest a relationship between GI bleeding and the use of sunitinib, there have been some case reports about VEGFIs, including sunitinib, related to GI perforation [64]. Recently, a study reported that the use of VEGFIs ≤ 3 months after SBRT significantly increased severe GI toxicity [65]. Therefore, clinicians should remember the possibility of higher risk of GI toxicity when IMRT with a hypofractionation regimen is combined with VEGFIs.

Another important issue about IMRT-related toxicity is the application of various toxicity criteria among studies, especially for hepatic toxicity. This causes changes in the incidence of hepatic toxicity, and makes it difficult to compare studies. Studies listed in Table 4 applied different toxicity criteria, including RILD alone, CTCAE or RTOG criteria alone, both RILD and CTCAE or RTOG criteria, as well as RILD, the change of CP class, and RTOG criteria. Historically, hepatic toxicity after RT has been documented as RILD. CP class and CTCAE have been used together to grade the prognosis of RILD, or used independently to assess hepatic toxicity [66]. RILD is useful in comparing results to historical studies, but rarely occurs in an era whereby advanced technology like IMRT or SBRT are used. The change of CP class is directly associated with patients’ prognosis, but some problems are associated with this measurement. Both the degree of ascites and encephalopathy are subjective assessments evaluated by performing physical examination alone, and the parameters are categorized with arbitrary cut-off points [67]. CTCAE criteria are more consist, but the occurrence of toxicity is not always related to clinical significance [68]. The following recent experts’ recommendations should be considered; Studies should separately record the incidence of classic and non-classic RILD, CP class should be recorded as well as any change in status of CP class after treatment, and the use of CTCAE criteria is advisable to promote consistency of reporting [69].

### New approaches

Because the GI organ is located near the liver, it is one of the important dose-limiting organs when RT is used to treat HCC. The classic method to minimize GI toxicity is to reduce target volume and decrease total dose using cone-down technique [59]. A more complex method is to use simultaneous integrated boost IMRT (SIB-IMRT), in which different doses are delivered to different targets at the same time. One study reported promising results of SIB-IMRT in HCC [70]. The following two dose-fractionation schemes were applied depending on the proximity of GI organ: (1) 41 patients in the low dose-fractionation group, with an internal target volume (ITV) < 1 cm from the GI organ who received 55 and 44 Gy in 22 fractions to planning target volume 1 (PTV1) and 2 (PTV2), and 12 patients in the high dose-fractionation (HD) group, with ITV ≥1 cm from the GI organ who received 66 and 55 Gy in 22 fractions to PTV1 and PTV2. There was no report of toxicity ≥ grade 3. Overall LCR and OS rates at 2 years were 67.3% and 54.7%, respectively. The HD group tended to show better objective RR (100% vs. 62%, *P* = 0.039), LCR at 2 years (85.7% vs. 59%, *P* = 0.119), and OS rate at 2 years (83.3% vs. 44.3%, *P* = 0.037). The authors suggested that the advantages of SIB-IMRT have been sustained in HCC although there is a risk of dose uncertainty resulting from liver motion during respiration.

Although surgical resection or transplantation are considered the standard treatments for HCC, less than 20% of patients are suitable for surgery, and recurrence rates can be as high as 25% per year after curative-intent surgery [71, 72]. To improve surgical outcome, several randomized trials investigated the role adjuvant treatment including systemic chemotherapy, HAIC, or TACE, but no adjuvant treatment has shown a therapeutic benefit, in terms of recurrence or OS after resection [73]. Recent technical advances in perioperative care have extended the surgical indications for HCC and patients with HCCs, that are adherent to the major vascular structures, are able to receive surgical resection. Recently, one study investigated the role of adjuvant IMRT in patients with HCCs close to major vessels [74]. In total, 181 patients were enrolled: 33 patients with narrow-margin (< 1 cm) after hepatectomy who received adjuvant s-IMRT (Group A), 83 patients with narrow-margin (< 1 cm) but who did not receive adjuvant RT (Group B), and 65 patients with wide-margin (≥ 1 cm) and who also did not receive adjuvant RT (Group C). Groups A and C showed significantly fewer intrahepatic marginal recurrences (*P* = 0.048) and diffuse recurrences (*P* = 0.018), and extrahepatic metastases (*P* = 0.038) than group B. The 3-year OS and disease-free survival rates were 89.1% and 64.2% in group A, 67.7% and 52.2% in group B, and 86.0% and 60.1% in group C. Based on this favorable outcome, the authors have embarked on an ongoing prospective phase II study with a larger patient cohort.

For locally advanced HCC, sorafenib, the multi-targeted tyrosine kinase inhibitor is recommended as the first-line treatment option from two phase III randomized trials [75–77]. Theoretically, a better treatment outcome is expected with a combination of IMRT and sorafenib for advanced HCC. One phase II study evaluated the efficacy and safety of IMRT with concurrent and sequential use of sorafenib for unresectable HCC with or without PVTT [78]. Total RT dose ranged from 40 Gy to 60 Gy (median: 50 Gy). Sorafenib was administered from the commencement of RT at a dose of 400 mg twice daily, and continued at the same dosage. They reported a promising 2-year OS rate of 32%, but patients experienced higher hepatic toxicity ≥ grade 3 than the above-mentioned studies. During IMRT, four patients (10%) experienced hepatic toxicity of grade 3. After IMRT, six patients (15%) developed hepatic toxicity ≥ grade 3, and three of them were fatal. They suggested that this combination should be used with caution. A phase I study of SBRT combined with sorafenib also reported high hepatic toxicity, including worsening of CP class in six patients (50%) with large-sized HCC [79]. Based on these studies, other sequences involving combination treatments with IMRT and sorafenib should be evaluated with the aim of reducing toxicity. There are inconsistent outcomes with pre-RT and post-RT use of sorafenib in preclinical studies. Li et al. [80] reported that sorafenib given 30 min before RT reduced the anti-proliferative effects of irradiation against HCC, whereas sorafenib given 24 hour after RT increased the anti-tumor effects against HCC *in vitro*. Yu et al. [81] found that there was inconsistent observation with pre-RT and post-RT use of sorafenib in two different cell lines. However, Chen et al. [82] suggested that pre-RT use of sorafenib could provide a better tumor growth inhibition than concurrent or post-RT use of sorafenib. Further studies are necessary to clarify the optimal sequence for use of this combination treatment.

## CONCLUSIONS

As the most advanced current radiotherapy technology, IMRT provides a precise and conformal dose distribution by using multiple beams with non-uniform intensity profiles. IMRT is divided into s-IMRT, VMAT, and h-IMRT according to beam delivery methods. Generally, IMRT techniques show better conformity to target volume coverage than 3DCRT for the treatment of HCC. Among IMRT techniques, VMAT and h-IMRT achieve a more homogenous dose distribution within a tumor than s-IMRT. Although the high dose region of the normal liver is smaller in IMRT than 3DCRT, the low dose region is increased in IMRT, and this increase is remarkable in h-IMRT or VMAT. Until present times, it has been unclear whether the increased low dose regions with IMRT techniques could be a risk factor for developing RILD. Additional studies are needed to confirm the safety of IMRT for treating HCC. In contrast, the sparing effect of non-liver OARs is beneficial in h-IMRT or VMAT.

Because of the differences in dosimetric advantages based on IMRT techniques, the choice of optimal IMRT technique should be personalized to the location of HCC and OARs. In HCCs < 6–8 cm, IMRT would be considered to spare the liver. In large HCCs or advanced HCC with PVTT, IMRT would be considered to spare non-liver OARs, especially in reducing high dose regions of gastroduodenum. In the clinical setting, a few studies have demonstrated the therapeutic benefit of IMRT for HCC in comparison to 3DCRT. Some studies utilizing IMRT reported promising treatment results. A new approach using SIB-IMRT provides another means of reducing GI toxicity. Adjuvant IMRT after surgical resection could reduce recurrence and increase survival in selected patients. A combination of sorafenib and IMRT resulted in a promising OS. However, many studies had small sample sizes, were retrospective in design, and had relatively short-term follow up periods. To validate the treatment outcomes of IMRT and obtain definitive conclusions, additional prospective studies in larger study populations will be necessary in the future.
